# Feasibility & Acceptability of Patient and Family Directed Active Music Making during Pediatric Bone Marrow Transplant Process

**DOI:** 10.3390/ejihpe12120131

**Published:** 2022-12-08

**Authors:** Annie Heiderscheit

**Affiliations:** Music Department, Augsburg University, Minneapolis, MN 55454, USA; heidersc@augsburg.edu

**Keywords:** active music making, patient directed music intervention, bone marrow transplant

## Abstract

(1) Background: Bone marrow transplant (BMT) is an aggressive and complex medical treatment for children with certain types of cancer and other diseases. The transplant process entails replacing the patient’s diseased bone marrow with the healthy marrow of a donor. During the course of treatment, patients are isolated in their room to reduce the risk of infection. Patient’s experience a variety of symptoms and side effects during the process including nausea, vomiting, diarrhea, mouth sores, fatigue, pain, discomfort, extreme muscle weakness, and emotional distress. Children often need a parent or caregiver present with them at all times throughout treatment. This process can cause significant stress and anxiety for the patient and their family. (2) Methods: This study explored the feasibility and acceptability of a patient and family directed active music making protocol during the BMT process. Ten patients, their parents, and family members participated in the study during the course of the BMT. (3) Results: Participants reported engaging in active music making 3–4 times per week and completed 121 journal forms reporting their music making experiences. They indicated using active music making to manage pain, discomfort, stress, anxiety, and boredom, foster relaxation and sleep, for enjoyment, and as a way to connect. (4) Conclusions: Patients, parents and family members reported feeling a sense of empowerment when engaged in making music to support their child or loved one.

## 1. Introduction

A bone marrow transplant (BMT) is an aggressive, curative medical procedure and process [1,2]. Since 2000, the number of bone marrow transplants (BMT) has steadily increased. In 2021, the Center for International Blood and Marrow Transplant Research (CIBMTR) reported over 20,000 BMT [1,3,4,5]. Throughout the course of the treatment process patients are confined to their room that is constructed with a specialized HEPA air filtration system designed to prevent and minimize infection [6,7,8,9,10,11,12]. Pediatric BMT patients can experience emotional and somatic distress, as well as physical discomfort throughout the treatment [6,7]. The pediatric BMT process necessitates a dedicated caregiver be present to provide emotional support and monitor and manage side effects of treatment to their child. Caregivers and family members often feel overwhelmed in the process as they juggle their child’s needs, work to understand and interpret medical information and processes, manage their work schedules, and address the needs of other children or family members [2,9,10]. Patients also experience a variety of challenges and symptoms during the BMT treatment process, which can include: fevers, chills, fatigue, extreme weakness, hives, nausea, vomiting, diarrhea, mouth sores, discomfort, and pain [2,3,4,5,6,7,13] further these symptoms can be complicated due to diminished emotional wellbeing, self-esteem, and social engagement [1,2,3,4,5,6,7,8,9,10,11,12,13,14]. The overwhelming nature of prolonged hospitalization and managing life-threatening complications necessitates integrating [7] a patient and family centered care approach to meet the unique needs of patients and their families [2] and allows them to be active agents in the treatment process [2,15,16,17,18,19]. This collaborative approach views the patient and their family as a resource, drawing upon their knowledge, and preferences to address their unique needs [17,18,20] and redefines the relationship of the patient, family, and healthcare providers, thus empowering patients and their families, as they determine the level to which they choose to participate in decision-making [20,21,22,23,24,25,26,27,28]. Further, a patient’s emotional well-being during the acute phase of their BMT treatment is a significant predictor of their overall social and physical functioning and parents and families play a critical role in helping manage anxiety, depression, and loneliness [13,29,30,31,32,33,34,35].

Music therapy has the potential to meet a variety of needs for patients during hospitalization as familiar music can provide a sense of comfort and security, and the innate and structure and flexibility in music has the capacity to allow for the unique responses and experiences of the patient [35]. Engaging with music, especially music that is familiar can help to normalize the hospital environment, provide an outlet for self-expression, and help to release tension and stress [36,37]. This multi-faceted nature of music and music therapy allows it to address patients’ psychosocial, emotional, and physical needs [38,39]. Previous music therapy studies indicate it helps to reduce anxiety, depression, distress, pain, fatigue, and fosters a relaxation response in reducing heart and respiratory rates [21,40,41,42,43,44,45,46,47,48,49,50]. Further, music therapy helps to promote supportive relationships, enables patients to engage in self-care, inspires playfulness and creativity, and fosters a sense of agency, control, and hope during the BMT process [16,22,51,52,53].

Implementation of patient directed music (PDM) interventions empowers patients to exercise their agency in determining what music they want to listen to, when they want to listen to it, and to decide how long they want to listen [54,55,56,57,58,59]. Patients initiating music listening showed significantly decreased levels of anxiety and significantly reduced levels of sedative exposure, frequency, and intensity [54]. Patients reported feeling empowered as they were able to choose what they preferred rather than a small selection of music provided to patients [57,58]. Patients and their families report that PDM listening helped improve sleep and provide a sense of comfort and enjoyment during and expressed gratitude and appreciation for a study that not only utilized music, but focused on the music preferences of the patient [60]. Research highlights the benefits of music therapy during the BMT process for patients and families and supports the use of patient directed music experiences. To date, research has not explored patient and family directed active music-making outside the context of music therapy sessions. This may be due to the fact that patients and families may need the musical structure and support the MT-BC provides, and that many musical instruments require training or experience to be able to play them. Therefore, structuring and designing music engagement experiences would be necessary to support patients and their families initiating active music making and to empower them to choose when they wanted to engage in making music.

## 2. Method

The aim of this feasibility and acceptability study was to explore the use of a patient and family directed active music making intervention during the BMT treatment process. The secondary aim was to understand what needs they were trying to address while engaging in active music making. A feasibility study design was selected in order to understand if study participants would deem it possible to engage with a patient and family active music-making intervention. The feasibility and acceptability of the active music-making intervention was evaluated based on patients and their families self-initiation of the intervention and their comments and feedback regarding why and how they used it and any challenges they encountered. The study protocol was designed by a board-certified music therapist (MT-BC), who is also licensed as a marriage and family therapist (LMFT). The active music-making protocol utilized instruments that were easily accessible to patients and families and did not require previous experience or training to be able to play.

### 2.1. Setting and Participants

The participants in this study included patients 6–13 years of age admitted to a 20-bed inpatient unit at a major pediatric medical center in the Midwest and were undergoing a bone marrow transplant and their family. Approval for the study was obtained through the Cancer Protocol Review Committee (CPRC) and the Institutional Review Board (IRB) at the University of Minnesota. Exclusion criteria included: (a) non-English or non-Spanish speaking and (b) lack of parent or caregiver engagement. Participants were not compensated for their participation in the study. Eligible patients and their parent(s)/caregivers were informed about the study by a research assistant. After completing the assent and consent forms, the MT-BC scheduled time to meet with them. After the patient and a parent completed the informed consent the MT-BC scheduled time to meet with them. Patients participated in their usual treatment throughout the study, up to 60 days.

### 2.2. Materials

In the initial meeting, the MT-BC delivered all the study equipment and materials, these included: a Reverie harp in a carrying case, stand for the harp, fifteen song sheets (for use with the Reverie harp), and various mallets and picks to use when playing the harp (including: dulcimer hammers, yarned mallets, three picks (soft, medium, and hard), two egg shakers, a 16″ Buffalo drum, and a Remo^TM^ Ocean Drum(manufactured by Remo, Inc., Valencia, CA, USA). These instruments were selected as they require little to no experience or training to play them, as well as to create a pleasing and musical sound.

The MT-BC also created and recorded twelve different instrumental music tracks that incorporated rhythm patterns and a variety of tempos played on various instruments (different types of drums, shakers, guitar, piano, etc.). These were developed in video format to allow participants to see and hear the rhythm patterns and play along. All of videos were uploaded and available to participants 24 h a day. They were able to access the videos via large screen television monitor in their room through the GetWellNetwork^TM^ (GetWellNetwork, Inc., Bethesda, MD, USA) The GetWell Network is an interactive patient communication system that allows patients to obtain scheduling information, order meals, received health education information, pharmacy records, as well as other information the health system make available [61].

Participants received an instructional sheet that provided step-by-step directions on how to access the play along videos on the GetWellNetwork^TM^. The MT-BC also demonstrated and showed the patients and parents/caregivers how to access the GetWell Network^TM^ to play along, as well as different ways to play the Reverie Harp, and how to utilize the song sheets. The MT-BC demonstrated how to play each instrument in various ways, how the harp could be played using the different mallets or strumming it with just a finger or using one of the picks and the different timbres these created. This allowed patients, parents, and family members to see and hear the unique sounds and music they could create. The MT-BC also engaged the patient and parents/family in a brief music making experience if they wanted. She also showed them how to access play along videos created for the study. She talked with them about when and how engaging in actively making music may be helpful and that it was their choice about when and how they wanted to use the instruments and make music, as well as who wanted to engage in making music. The MT-BC created and provided journal forms for patients and family members to write about who was engaged in the process, when, how, and why they were using the Reverie harp and the other instruments, and describe their experiences of making music together.

All the study instruments and materials remained in the patient’s room throughout their course of treatment and inpatient stay. The MT-BC met with the patient and their parents/family twice weekly to check in and see if they had any questions, needed assistance, tune the Reverie harp, and ensure that all the materials were functioning properly. She checked in and talked with the patient and their family about if, and/or how they were using the Reverie harp, instruments, and video tracks and to also collect their journal forms (included in Supplemental Table S1).

Throughout the course of the study 13 patients and their families were provided information about the study and 10 of those completed the informed consent. Table 1 includes patient demographic information, including age, gender, details about parent and family members engaged with the patient during treatment, and the patient’s length of stay. Study participants ranged from 7 to 13 years of age with a mean age of 9 years. Sixty percent of participants were female, 60% white, 20% Hispanic, and 20% black. Ninety percent of study participants were diagnosed with some form of cancer, of which 60% were leukemia. The length of stay for participants ranged from 27 to 46 days with a mean of 35.5 days.

### 2.3. Analysis

Journal forms participants and their family’s completed, as well as experiences they communicated to the MT-BC during the weekly visits, were reviewed to identify why they were choosing to engage in actively making music, based on their words and the descriptions of their music making experiences. The MT-BC served as the interventionist in this study and as the reviewer journal forms in the analysis process. In this iterative analysis process, these emergent themes were then clustered and grouped in categories with which they were similarly oriented. These categories were examined, searching for points and patterns of connection and then developed into subordinate themes. The emergent themes were cross-referenced with the 121 journal forms until all the data had been extracted. The themes about the reasons why they engaged in active music making were revised until they best reflected the patterns of the emergent themes and the data from the journal forms and detailed written notes maintained by music therapist from weekly meetings [62]. These themes and sub ordinate themes were reviewed by a second reviewer and were also provided and reviewed by study participants for their feedback to ensure they were reflective of their experiences.

## 3. Results

Study participants reported that throughout the study, 80% of the time they had multiple family members engaged in actively making music with them, this included parents, siblings, and grandparents. Parents and caregivers reported that 30% of the time, they played music when the patient felt weak, too tired, or nauseous to actively engage in making music. While 121 journal forms were collected, three patients and families did report that they forgot to fill out seven journal forms. This resulted in two families missing three forms and one family missing one form. Based on the forms collected, patients and their families engaged in actively making music between 3 to 4 times per week.

Patients, parents, siblings, and family members reported on the journal forms and during check-ins with the MT-BC, when and why they were engaging in actively making music. They reported many multiple and different reasons for engaging in active music making that ranged from managing symptoms associated with the BMT treatment process, managing the challenges prolonged room isolation, and as a means of engaging with one another. Table 2 provides an overview of the patient, the family members with whom they engaged with in actively making music, and the range of reasons they engaged in active music making.

Participants families reported that active music making allowed them to adapt the experience to meet changing needs, as well as different needs simultaneously. On journal forms and talking with the MT-BC during weekly visits they shared that they engaged in actively making music for various reasons and to manage and address different physical needs resulting from the treatment course (fatigue, nausea, pain, discomfort), and psychosocial needs (manage stress, anxiety, boredom, mood, foster connection, and for enjoyment). Analysis of the journal forms and notes from conversations that occurred during weekly visits with MT-BC resulted in six sub themes and three main themes emerging. The three themes include: choice, music as a resource, and empowerment. Table 3 includes the sub themes of choice and provides an overview of what patients and families related to these. They reported that while they were not able to make many choices during the BMT treatment process, they could choose to use music. They could choose to use music to manage different symptoms and they had choices regarding what instruments to use and ways to use them.

The sub themes of music as a resource are explicated in Table 4 and include accessible, discovering ability to make music, and a way to cope. Patients and families reported that the choice instruments and various tools (song sheets and play along tracks) made actively making music accessible to them, allowed them to explore and discover the ability to make music, and use music as a means of coping. They were able to utilize music as a way to address different needs and their willingness to explore the use of music allowed them to discover their capacity to make use of music as a resource. Table 5 includes the sub themes of empowerment which include patients and families ability to act, experiencing a sense of agency, and being able to exert control. Patients and families indicated that being able to decide if, when, and how to engage in making music afforded them the opportunity to feel a sense of agency and make their own decisions in these moments.

Patients and families shared quotes about their experiences of actively making music through the BMT process. The supplemental material Table S2 includes direct quotes from patients and their family members that describe their music making experiences. These quotes range from referring to specific moments when they engaged in active music making or provide details about their overall experiences. The quotes provide information regarding why patients and their families chose to engage in actively making music, what occurred during those experiences, and what they discovered in the process of making music together.

## 4. Discussion

The aim of this study was to explore the feasibility and acceptability of a patient directed active music making with patients and their families during the BMT process. Music-based interventions are more widely being utilized for patients and their families in various healthcare settings and address challenges related to the treatment process and symptom management [56]. The results from this feasibility and acceptability study align with findings from music therapy research that indicate it can help address many of the symptoms that patients experience during the BMT process, such as, nausea, fatigue, pain, discomfort, and emotional distress [45,46,47,48,58,63]. A key difference in the current study is the use of a patient and family directed active music making protocol, which allows patients and the family to utilize active music making whenever they want or need the intervention. The sample size for this study was small as the study was designed to test feasibility and acceptability, as a result the findings of this study are limited should not be generalized and presumed to represent all patients and families experiencing hospitalization.

Patients and their families report of the use of the music-based intervention and their frequency of use indicate that it is a feasible and acceptable intervention in the BMT process to cope with their prolonged hospitalization and isolation, or with challenges symptoms [58,63]. Patients and their families reported actively engaging in making music together not only addressed many different needs, it also addressed different needs for each family member simultaneously. Additionally, they were able to engage in making music in different ways to meet their needs as they changed throughout the treatment process [23,64]. The flexible way in which they were able to engage in and with the music allowed them to music in a non-prescriptive way and to consider what they needed in the moment, allowing them to explore and discover how music could meet those needs, mirroring the flexible manner that an MT-BC would employ while responding to the patient in a music therapy session [21,22].

While patients and families in the study reported feeling empowered by actively engaging in music making together, they indicated they had to work to overcome their judgements and insecurities about not being trained musicians. Encouragement from the MT-BC, playing familiar songs (provided on the song sheets), along with continued engagement helped to mitigate this. They shared they benefited from learning from the MT-BC how music and active music making maybe to them helpful during the BMT process. They reported that learning about how actively making music can be beneficial fostered their motivation to seek out opportunities to engage with music including improvising music or songs. They also recognized that when they were overwhelmed or stressed in the treatment process, the information and reminders from the MT-BC about the benefits of music making, served as opportunities to ask questions, check-in, and helpful prompts to engage with music. learn and integrate new knowledge about the benefits of music and active music making. Further, the MT-BC validated their experiences with music, which helped foster their sense of competency in actively making music and to recognize music as a resource

## 5. Conclusions

Patient and family’s active engagement in the care process is a marker for improved health outcomes. Actively engaging patients and their families in creating and making music during their treatment process empowers them to make decisions about when and how to use music as a part of their process. Patients and their families indicate that they chose to use active music-making helps to meet various needs throughout the BMT process and that future research should focus on quantifying the impact of patient and directed active music making. This should include randomized controlled trials that aim to evaluate outcomes and standardized measures related to patient and family coping, quality of life, pain, and symptom management. Due to the key role that parents and family members play in the patient’s treatment process, it is important that interventions are inclusive of addressing their needs and wellbeing as well. Further, while there is limited research examining the economic impact of patient directed music listening, exploring the cost-effectiveness of a patient and family directed active music making intervention is also warranted [65].

Hospitals can work to integrate patient and family directed active music-making interventions by collaborating and consulting with a trained and credentialed music therapist. It is evident from the patient and families experiences they need structure and music resources the MT can help to create to be able to engage in this type of intervention. Further, they benefit from understanding how music and active music-making can be a resource. It is important to develop and adapt the intervention to meet the musical capacities of the patients and families, as well as consider music preferences and cultural considerations.

## Figures and Tables

**Table 1:** Patient demographic information.

Age	Gender	Ethnicity	Diagnosis	Length of Stay	Parent/Family Participants
8	F	White	Leukemia	37 days	Mom and two younger siblings (ages 6 and 4)
12	M	White	Neuroblastoma	46 days	Mom and Dad
9	M	Black	Leukemia	39 days	Mom and Grandmother
11	F	White	Hurler syndrome	33 days	Mom and brother (age 9)
13	F	White	Leukemia	29 days	Dad and sister (age 10)
7	M	Hispanic	Rhabdomyosarcoma	41 days	Dad
9	M	White	Leukemia	27 days	Mom
10	F	Black	Leukemia	31 days	Mom, Dad and brother (age 4)
12	F	White	Lymphoma	38 days	Mom, Dad, Grandmother, and Grandfather
8	F	Hispanic	Leukemia	34 days	Mom and sister (age 11)

**Table 2:** Reasons reported for engaging in actively music making.

Patient Age	Family Involved Active Music Making	Reasons for Engaging in Active Music Making
8	Mom and two siblings	Enjoyment, relaxation, manage stress and anxiety, to soothe patient, and help patient sleep
12	Mom and Dad	Distraction, improve mood, manage discomfort and nausea, and relaxation
9	Mom and Grandmother	Curiosity about the instruments, enjoyment, foster relaxation, means distraction, and manage discomfort
11	Mom and brother	Provide distraction, foster relaxation, and means of enjoyment, way to engage with one another
13	Dad and sister	Engaging with each other, foster relaxation, and to help the patient sleep
7	Dad	Manage boredom, improve mood, relaxation, and manage discomfort and nausea
9	Mom	Manage stress and anxiety, distraction from boredom, manage pain and discomfort
10	Mom, Dad and brother	Enjoyment, manage anxiety, foster relaxation, and manage boredom
12	Mom, Dad, Grandmother, and Grandfather	Engaging with each other, enjoyment, foster relaxation, manage anxiety, and distract from discomfort
8	Mom and sister	Provide distraction, manage discomfort and nausea, and foster relaxation

**Table 3:** Sub themes of Choice.

Subthemes	Patients and Families Reported
Decision to use music	Making music was one decision they felt they could make for ourselves in the treatment process
	Independently able to make a choice to use music
	Able to choose to use music whenever wanted or needed
Manage symptoms	Discovered music could be used to address symptoms rather than to only have to rely on medication
	Use of music to manage many different symptoms
	Music addressed different symptoms for each family member simultaneously
Different ways to engage with music	Various instruments and tools provided many different ways to engage with music
	Possible for some family members to play music while others listened
	Continued to learn new and different ways to make music from MT

**Table 4:** Sub themes of Music as a resource.

Sub Themes	Patients and Families Reported
Accessible	Instruments and play along tracks were accessible 24 h a day
	MT-BC provided information and instruction to make music accessible
	Learning different ways to use the instruments and music increased capacity to use it
Discovering ability to make music	Overcoming doubt and judgement about one’s musical ability
	Learning different ways to play the instruments helped them feecomfortable and more confident with actively making music
	Using play along tracks on the GetWell Network^TM^ provided structure needed to actively engage in making music
A way to cope	Music engagement adapted to address different needs of patientand family simultaneouls
	Helped manage different challenges in the BMT process (isolation, boredome, stress, anxiety, and symptoms)
	A collaborative means of coping as a family unit

**Table 5:** Sub themes of Empowerment.

Sub Themes	Patients and Families Reported
Ability to act	Access to the instruments and other music resources
	at all times affoded them the ability to use the music
	When treatment felt unmanageable or overwhelming active engage with the music was still possible
	Integrated active music making into other aspects of treatment that were especially challenging
Sense of agency	Making music was often the only area of treatment that provided choice
	Change and adapt the music to what they needed it to be
	Discovered capacity to utilize music as a resource to cope
Exert control	Ablility to choose to make music afforded feeling a sense of control
	Music was integrated with medication to enhance symptom management or used in lieu of medication
	When things felt too difficult to manage making music provided a means of managinig what felt out of control

## Data Availability

Not applicable.

## Supplementary Materials

The following supporting information can be downloaded at: https://www.mdpi.com/article/10.3390/ejihpe12120131/s1, Supplemental Material Table S1. Journal Form and Supplemental Material Table S2. Quotes about actively making music experiences.

## References

[1] D’Souza A., Lee S., Zhu X., Pasquini M. (2017). Current use and trends in hematopoietic cell transplantation in the United States. Biol. Blood Marrow Transplant..

[2] Runaas L., Hanauer D., Maher M., Bischoff E., Fauer A., Hoang T., Mynaco A., Snakaran R., Gupta R., Seyedsalehi S. (2017). BMT Roadmap: A user-centered design health information technology tool to promote patient-centered care in pediatric hematopoietic cell transplantation. Biol. Blood Marrow Transplant..

[3] Kaziunas E., Buyuktur A., Ones J., Choi S., Hanauer D., Ackerman M. Transition and reflection in the use of health information: The case of pediatric bone marrow transplant caregivers. Proceedings of the Association for Computing Machinery (ACM) on Computer Supported Cooperative Work and Social Computing.

[4] Riedell P., Hamadani M., Ahn K., Litovich C., Murthy G., Locke F., Brunstein C., Merryman R., Stiff P., Pawarode A. (2021). Outcomes and utilization trends of front-line autologous hematopoietic cell transplantation for mantel cell lymphoma. Transplant. Cell. Ther..

[5] Auletta J., Kou J., Chen M., Shaw B. Current Use and Outcome of Hematopoietic Stem Cell Transplantation: CIBMTR Summary Slides. http://www.cibmtr.org.

[6] Phipps S. (2002). Reduction of distress associated with paediatric bone marrow transplant: Complementary health promotion interventions. Pediatric Rehabil..

[7] Phipps S., Dunavant M., Garvie P., Lensing S., Rai S. (2005). Acute health-related quality of life in children undergoing stem cell transplant: I. Descriptive outcomes. Bone Marrow Transpl..

[8] Bishop M., Curbow B., Springer S., Lee J., Wingard J. (2011). Comparison of lasting life changes after cancer and BMT: Perspectives of long-term survivors and spouses. Psychoncology.

[9] El-Jawahri A., Traeger L., Kuzmuk K., Eusebio J., Vandusen H., Shin J., Jackson V. (2015). Quality of life and mood of patients and family caregivers during hospitalization for hematopoietic stem cell transplantation. Cancer.

[10] Felder-Puig R., Peters C., Matthes-Martin S. (1999). Psychosocial adjustment of pediatric patients after allogeneic stem cell transplantation. Bone Marrow Transpl..

[11] Felder-Puig R., Di Gallo A., Waldenmair M. (2006). Health related quality of life of pediatric patients receiving allogeneic stem cell or bone marrow transplantation: Results of a longitudinal, multi-center study. Bone Marrow Transpl..

[12] Packman W., Weber S., Wallace J., Bugescu N. (2010). Psychological effects of hematopoietic SCT on pediatric patients, siblings, and parents: A review. Bone Marrow Transpl..

[13] Phipps S., Dunavant M., Lensing S., Rai S. (2005). Psychosocial predictors of distress in parents of children undergoing stem cell or bone marrow transplantation. J. Pediatric Psychol..

[14] Von A., Spath M., Nielsen A., Fife B. (2016). The caregiver’s role across the bone marrow transplantation trajectory. Cancer Nurs..

[15] Yates G., Beckman N., Voss M., Anderson M., Silverman M. (2018). Caregiver perceptions of music therapy for children hospitalized for a blood and marrow transplant: An interpretivist investigation. Glob. Adv. Health Med..

[16] Streisand R., Rodrigue J., Houck C., Graham-Pole J., Berlant N. (2000). Brief report: Parents of children undergoing bone marrow transplantation: Documenting stress and piloting a psychological intervention program. J. Pediatric Psychol..

[17] Rawson J., Moretz J. (2016). Patient- and Family-Centered Care: A Primer. J. Am. Coll. Radiol..

[18] Park M., Giap T., Lee M., Jeong H., Jeong M., Go Y. (2018). Patient- and family-centered care interventions for improving the quality of health care: A review of systematic reviews. Int. J. Nurs. Stud..

[19] Lusk J.M., Fater K. (2013). A concept analysis of patient-centered care. Nursing Forum.

[20] Johnson B. (2013). Promoting Patient- and Family-Centered Care Through Personal Stories. Acad. Med..

[21] Aasgaard T. (2001). An ecology of love: Aspects of music therapy in the pediatric oncology environment. J. Palliat. Care.

[22] O’Neill B., Pavlicevic M. (2003). Exploring a role for music therapy with children undergoing bone marrow transplantation at Great Ormond Street Hospital, London. Br. J. Music. Ther..

[23] Dun B. Journeying with Olivia: Bricolage as a Framework for Understanding Music Therapy in Paediatric Oncology. http://www.voices.no/mainissues/mi40007000229.php.

[24] Gooley T., Chien J., Pergam S., Hingorani S., Sorror M., Boeckh M. (2010). Reduced mortality after allogeneic hematopoietic-cell transplantation. N. Engl. J. Med..

[25] Fife B., Von Ah D., Weave M., Yang Z., Stump T., Farag S. (2017). Preliminary efficacy of a brief family intervention to prevent declining quality of life secondary to parental transplantation. Bone Marrow Transplant..

[26] Keogh F., O’Riordan J., McNamara C., Duggan C., McCann S. (1998). Psychosocial adaptation of patients and families following bone marrow transplantation: A prospective, longitudinal study. Bone Marrow Transpl..

[27] Langer S., Abrams J., Syrjala K. (1998). Caregiver and patient marital satisfaction and affect following hematopoietic stem cell transplantation: A prospective, longitudinal investigation. Psychooncology.

[28] Fife B., Monahan P., Abonour R., Wood L., Stump T. (2009). Adaptation of family caregivers during the acute phase of adult BMT. Bone Marrow Transplant..

[29] Applebaum A., Bevans M., Son T., Evans K., Hernandez M., Giralt S., Du Hamel K. (2016). A scoping review of caregiver burden during allogeneic HSCT: Lessons learned and future directions. Bone Marrow Transplant..

[30] West C., Bell J., Woodgate R., Moules N. (2015). Waiting to return to normal: An exploration of family systems intervention in childhood cancer. J. Fam. Nurs..

[31] West C., Dusome D., Winsor J., Rallison L. (2020). Falling down the rabbit hole: Child and family experiences of pediatric hematopoietic stem cell transplant. Qual. Health Res..

[32] Jobe-Shields L., Alderfer M., Barrera M., Vannatta K., Currier J., Phipps S. (2009). Parental depression and family environment predict distress in children before stem cell transplantation. J. Dev. Behav. Pediatrics.

[33] Wennström B., Johansson A., Kalabic S., Loft E.S., Skullman S., Bergh I. (2020). Patient experience of health and care when undergoing colorectal surgery within the ERAS program. Perioper. Med..

[34] Reiche E., Nunes S., Morimoto H. (2004). Stress, depression, the immune system, and cancer. Lancet Oncol..

[35] Robb S.L. (2003). Designing music therapy interventions for hospitalized children and adolescents using a contextual support model of music therapy. Music. Ther. Perspect..

[36] Hadley S. (1996). A rationale for the use of songs with children undergoing bone marrow transplantation. Aust. J. Music. Ther..

[37] Kennelly J. (2001). Music therapy in the bone marrow transplant unit: Providing emotional support during adolescence. Music. Ther. Perspect..

[38] Davidson B. (2001). Music therapy and childhood cancer: Goals, methods, patient choice, and control during diagnosis, intensive treatment, transplant and palliative care. Music. Ther. Perspect..

[39] Weaver C., Dwiggins A., McCormick K., Richart J., Saad J. (2017). A retrospective examination of issues addressed during music therapy sessions with bone marrow transplant recipients. Biol. Blood Marrow Transpl..

[40] Boldt S. (2017). The effects of music therapy on motivation, psychological well-being, physical comfort, and exercise endurance of bone marrow transplant patients. J. Music. Ther..

[41] Cassileth B., Vickers A., Magill L. (2003). Music therapy for mood disturbance during hospitalization for autologous stem cell transplantation: A randomized controlled trial. Cancer.

[42] Sahler O., Hunter B., Liesveld J. (2003). The effect of using music therapy with relaxation imagery in the management of patients undergoing bone marrow transplantation. Altern. Ther. Health Med..

[43] Rosenow S., Silverman M. (2014). Effects of single session music therapy on hospitalized patients recovering from a bone marrow transplant: Two studies. Arts Psychother..

[44] Robb S., Burns D., Stegenga K., Haut P., Monahan P., Meza J., Stump T., Cherven B., Docherty S., Hendricks-Ferguson V. (2014). Randomized clinical trial of therapeutic music video intervention for resilience outcomes in adolescents/young adults undergoing hematopoietic stem cell transplant: A report from the Children’s Oncology Group. Cancer.

[45] Tucquet B., Leung M. (2014). Music therapy services in pediatric oncology: A national clinical practice review. J. Pediatric Oncol. Nurs..

[46] Imran S., Moosabba M., Ancheril A. (2016). Music therapy for improving bio-physiological and psychological outcomes in patients with cancer: A review of literature. Int. J. Med. Pediatrics Oncol..

[47] Ortiz G., O’Connor T., Carey J., Vella A., Paul A., Rode D., Weinberg A. (2019). Impact of a child life and music therapy procedural support intervention on parental perception of their child’s distress during intravenous placement. Pediatric Emerg. Care.

[48] Reimnitz L., Silverman M. (2020). A randomized pilot study of music therapy in the form of patient-preferred live music on fatigue, energy and pain in hospitalized adult oncology patients on a blood and bone marrow transplant unit. Arts Health.

[49] Facchini M., Ruini C. (2021). The role of music therapy in the treatment of children with cancer: A systematic review of literature. Complement. Ther. Clin. Pract..

[50] Barry P., O’Callaghan C., Wheeler G., Grocke D. (2010). Music therapy CD creation for initial pediatric radiation therapy: A mixed methods analysis. J. Music. Ther..

[51] O’Callaghan C., Dun B., Baron A., Barry P. (2013). Music’s relevance for children with cancer: Music therapist’s qualitative clinical data-mining research. Soc. Work. Health Care.

[52] Verstegen A., Silverman M. (2018). Effects of music therapy on mood and pain with patients hospitalized for bone marrow transplantation: A randomized effectiveness pilot study. J. Creat. Ment. Health.

[53] Fredenburg H., Silverman M. (2014). Effects of music therapy on positive and negative affect and pain with hospitalized patients recovering from blood and marrow transplantation: A randomized effectiveness study. Arts Psychother..

[54] Chlan L., Weinert C., Heiderscheit A., Tracy M.F., Skaar D., Guttormson J., Savik K. (2013). Effects of patient directed music intervention on anxiety and sedative exposure in critically ill patients receiving mechanical ventilatory support. J. Am. Med. Assoc..

[55] Chlan L., Heiderscheit A., Lindquist R., Snyder M., Tracy M.F. (2022). Music Intervention. Complementary and Alternative Therapies in Nursing.

[56] Heiderscheit A., Chung Ong K. (2021). Non-pharmacological management of symptoms during mechanical ventilation and chronic obstructive pulmonary disease in critical care: Patient directed music listening. Chronic Obstructive Pulmonary Disease—A Current Conspectus.

[57] Heiderscheit A., Chlan L., Donley K. (2011). Instituting a music listening intervention for critically ill patients receiving mechanical ventilation: Exemplars from two patient cases. Music. Med..

[58] Heiderscheit A., Zambonini J.P., Andonian K., Manno J., Wheeler B., Viega M., Dos Santos A. (2022). Music Therapy for Procedural Support. Music Therapy Handbook.

[59] Heiderscheit A., Allen J. (2013). Music therapy in Surgical and Procedural Support for Adult Medical Patients. Guidelines for Music Therapy with Adult Medical Patients.

[60] Tracy M., Staugitis A., Chlan L., Heiderscheit A. (2015). Perceptions of Patients and Families who Received a Music Intervention during Mechanical Ventilation. Music. Med..

[61] Delaney M. (2018). GetWell Network Integrates IoMT Technology to Help Providers Optimize Patient Engagement. HealthTech.

[62] Braun V., Clarke V. (2013). Successful Qualitative Research.

[63] Heiderscheit A., Johnson K., Chlan L. (2022). Analysis of preferred music mechanically ventilated ICU patients enrolled in a randomized controlled trial. J. Integr. Complementary Med..

[64] Lane D. (1993). Music therapy: Gaining an edge in oncology management. J. Oncol. Manag..

[65] Chlan L., Heiderscheit A., Skaar D., Neidecker M. (2018). Economic evaluation of patient directed music intervention compared to usual care on costs in ICU patients receiving mechanically ventilatory support. Crit. Care Med..

