# Supplementary material for: Feasibility & Acceptability of Patient and Family Directed Active Music Making during Pediatric Bone Marrow Transplant Process
Source: Eur J Investig Health Psychol Educ. 2022 Dec 8;12(12):1867–77. doi: 10.3390/ejihpe12120131 (PMC9777901; doi:10.3390/ejihpe12120131)
Supplement: Supplementary file 1 [file ejihpe-12-00131-s001.zip › Table S1.pdf]

Table S1 Journal Form

Participant ID #: ________________

Journal Form

Date: _________________________________

Check any of the following that describe how you engaged in actively making-music today:

___ Played for my child
___ Played with my child
___ Played with other family members: Indicate who: ____________________________
___ Played with staff
___ Some other way: Please indicate: ____
____
____
____

Please share your experiences, thoughts, feelings about engaging with actively making-music. You can include any aspect of our experience such as why you chose to make music, how it made you feel to make music, what was challenging about making music, if or how it helped to make music, or what you learned from making music.

____
____
____
____
____
____
____
____
____
____
____
____
____
____
____
____
____

Music therapist will collect complete journal forms each week.
