# Supplementary material for: Feasibility & Acceptability of Patient and Family Directed Active Music Making during Pediatric Bone Marrow Transplant Process
Source: Eur J Investig Health Psychol Educ. 2022 Dec 8;12(12):1867–77. doi: 10.3390/ejihpe12120131 (PMC9777901; doi:10.3390/ejihpe12120131)
Supplement: Supplementary file 1 [file ejihpe-12-00131-s001.zip › Table S2.pdf]

Supplemental Table S2

Quotes about actively making music experiences

Patient age	Family member	Quotes from patients and families
8	Mom and two siblings	*Mom:* "My daughter struggles with anxiety. I was able to start playing the harp the other day and she began to calm. Eventually, we began to play the harp together. She was able to move from this anxious state to a calm state."
12	Mom and Dad	*Mom and Dad*: "We often felt powerless when he was feeling so nauseous. We did not know how to help. One day we decided to create and play some music for him to see if that might be helpful. We felt uneasy at first, but as we continued to play, we noticed that he seemed more relaxed, we felt more relaxed, and we were able to rest and sleep. We felt less anxious and more relaxed as well."
9	Mom and Grandmother	*Grandmother:* "When I come visit, I often feel unable to help him. If he is tired or feeling nauseous, he does not have the energy to play a game. I found that we could make music together and he could engage playing adjusting his playing how he was feeling and I could follow and support him. Playing calming and soothing music together often helped him feel better." *Mom:* "I enjoyed being able to trying something new during this challenging process. We had fun playing different rhythms with the videos from the GetWell Network". Patient: "I liked being able to make and create music. I was able to create and play music that expressed different feelings and my grandma and mom would guess the feeling."
11	Mom and brother	*Mom:* "Even under normal circumstances it can be difficult for an 11-year-old girl to get along with her 9-year-old brother. Feeling nauseous, tired and uncomfortable do not make this any easier for her or any of us. We were all able to find moments of respite, connection, and peace when we make music together. Her brother even found enjoyment in playing something to help his sister rest and relax."
13	Dad and sister	*Patient:* "I did not like when my dad and sister would have to leave in the evening. I always felt sad and lonely at night when they weren't there. Sometimes we would make music together before they left and that would help me to feel less lonely, relax, and fall asleep." *Dad:* "I often felt overwhelmed in trying to balance work and the needs of both my daughters during the BMT treatment process. I engaged with them in making music because I could see how much they enjoyed it. I discovered that it was not only helpful for them, but it also helped me relax and manage stress."
7	Dad	*Dad:* "I came in yesterday and my son was very irritable and feeling nauseous. When I tried to talk with him, he would just snap or hide under the blanket. I eventually just picked up the harp and began to play. He came out from under the blanket and eventually just fell asleep. I thought I was playing for him, but I realized when I stopped playing that I needed it too and that I was really playing for both of us."
9	Mom	*Mom:* "I played the harp the other day when my son was not feeling well. He quickly realized and fell asleep. I found myself transported by the music. I felt like I was in Japan."
10	Mom, Dad, and brother	*Mom:* "It was a difficult time as our 4-year-old wanted to see his sister. We tried to limit the visits as we felt it was too much for him to understand. When he did visit, he was very curious about the instruments and he wanted to make music. We discovered that making music together made this a very different experience than we had anticipated. We all enjoyed the experience of playing music and walked away from those visits feeling more connected as a family."

12	Mom, Dad, Grandmother, and Grandfather	*Mom:* "I found that my husband and my dad initially felt uncomfortable being in the room. They wanted and needed something to do to be helpful. When the MT reminded us of how actively making music can be helpful, I realized this was something they could do to help. After they moved past their own uneasiness of trying something new, they discovered how it helped her (patient) and from then on, they asked her if she wanted to make music together or if she wanted them to play music for her."
8	Mom and sister	*Patient:* "I played the harp while my sister played the ocean drum. We had fun exploring the different sounds we could make." *Mom:* "It was so nice to see them engaging with each other in such a playful and normal way here in the hospital."
